# Supplementary material for: The Construction and Exploration of a Comprehensive MicroRNA Centered Regulatory Network in Foxtail Millet (Setaria italica L.)
Source: Front Plant Sci. 2022 May 6;13:848474. doi: 10.3389/fpls.2022.848474 (PMC9121102; doi:10.3389/fpls.2022.848474)
Supplement: Supplementary file 6 [file Data_Sheet_6.zip › Sit-miR396c_Seita.5G318400.1_3005_TPlot.pdf]

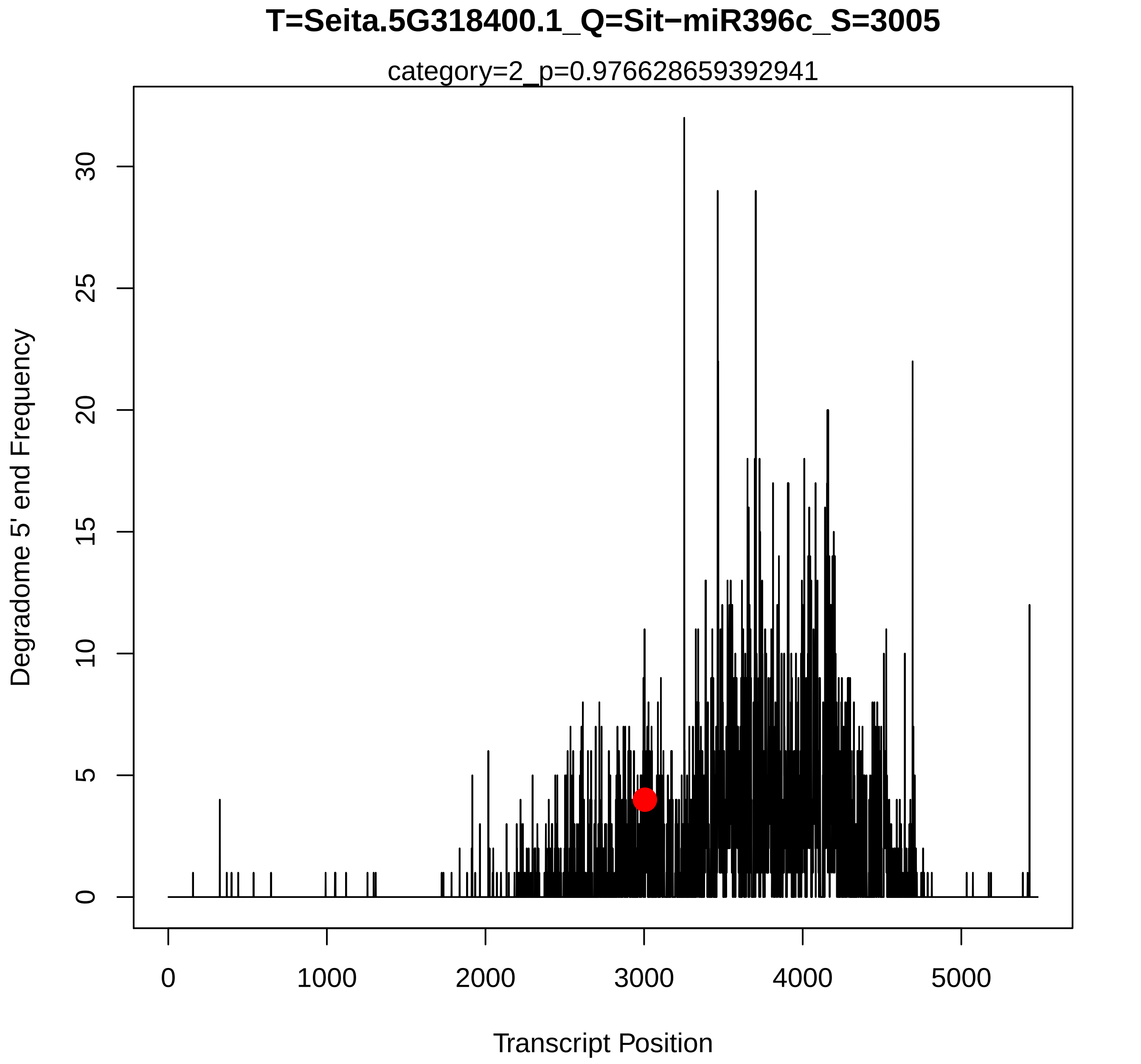
T=Seita.5G318400.1_Q=Sit−miR396c_S=3005
category=2_p=0.976628659392941
Degradome 5' end Frequency
0
5
10
15
20
25
30
0
1000
2000
3000
4000
5000
Transcript Position
